# Exploring stigma of physical health conditions across low and lower-middle income countries: a secondary analysis of a global systematic review

**DOI:** 10.3389/fpubh.2026.1912141

**Published:** 2026-10-14

**Authors:** Bhargavi Ram, Varun Elagandula, Lara Counts, Luke Zabotka, Anneliese Williams, Kayla Wynja, Sara Malone, Dylan E. Graetz

**Affiliations:** 1 Department of Global Pediatric Medicine, St. Jude Children’s Research Hospital, Memphis, TN, United States; 2 School of Public Health, Washington University in St. Louis, St. Louis, MO, United States; 3 Department of Public Health, Purdue University, West Lafayette, IN, United States

**Keywords:** global health, low-and lower-middle-income countries, secondary analysis, stigma, stigma characteristics

## Abstract

**Introduction:**

Stigma undermines health equity by impeding engagement in care and worsening outcomes. Systematic reviews of stigma are primarily from high-income countries, focus on specific conditions, especially mental illness, and employ heterogeneous stigma measures. Despite bearing a disproportionate burden of disease, low-income (LIC) and lower-middle-income (LMIC) countries remain underrepresented in existing reviews. This study characterizes stigma definitions, measures, impacts, and interventions in physical health conditions across LICs and LMICs.

**Methods:**

This secondary analysis included 137 publications, previously identified through a global systematic review, reporting on patient or caregiver stigma of physical health conditions among LIC/LMIC populations. Data on definitions, measurements, and interventions were utilized from a primary review. Two reviewers extracted supplemental data regarding the study’s conceptualization and outcomes and conducted a quality appraisal.

**Results:**

Among the 137 studies (32 LIC, 105 LMIC), 75.2% used cross-sectional designs, 67.2% focused on adults, and 75.9% included populations with communicable diseases, including HIV, TB, and leprosy. Stigma definitions and measures varied widely, with uneven coverage of stigma dimensions. Negative mental health outcomes were commonly associated with stigma related to physical conditions (57.7%). Only 13.1% of studies evaluated stigma interventions, primarily including social support or health education, with heterogenous intervention designs, outcome definitions, and follow-up periods. Our quality appraisal demonstrated limited use of population-validated and culturally adapted stigma tools.

**Conclusion:**

Stigma research in LIC/LMICs has primarily been conducted in middle-income countries and focused on adults with communicable diseases, leaving gaps for stigma research in non-communicable diseases and pediatric populations. Future work should prioritize development of culturally adapted stigma frameworks and measures to improve patient outcomes in LIC/LMICs.

**Systematic review registration:**

https://www.crd.york.ac.uk/PROSPERO/view/CRD42023433176.

## Introduction

Stigma is the co-occurrence of stereotyping, discrimination, labeling, and status loss (1, 2). It contributes to negative health outcomes by exacerbating mental and physical health burdens and is a barrier to treatment engagement and adherence. The pervasive impact of stigma makes it a critical determinant of health globally, especially in settings with constrained health systems.

Systematic reviews with data largely from high-income countries (HICs) have explored stigma across various health conditions and provided an evidence base for psychosocial interventions. These reviews have highlighted the effects of stigma and identified key factors that could affect stigma, such as ethnicity, age, and co-morbid conditions (3–9). However, most stigma research has focused on specific disease categories and populations, such as mental health disorders, Human Immunodeficiency Virus (HIV), and cancer (8–11). The studies described in these reviews use multiple different frameworks, measures, and intervention strategies to reduce stigma. Prior work has highlighted the need for more standardized approaches to studying stigma, which would enable widespread application.

Despite efforts to understand stigma globally, there is a gap in understanding how stigma is studied and conceptualized in low-income countries (LICs) and lower-middle income countries (LMICs) (8, 12–14). Evidence from HICs may not adequately capture the dynamics of stigma in LICs and LMICs, where differing social norms, cultural contexts, and structural conditions, including health system constraints, shape stigma experiences (15). Additionally, existing stigma research under-represents non-communicable diseases (NCD) that are common, chronic, visible, and socially misunderstood within these settings (16–18). The aim of our study is to characterize definitions and approaches to measuring stigma across physical health conditions in LIC and LMIC studies, evaluate methodological rigor, examine associations with stigma, and highlight priorities for future studies on stigma and stigma-reduction strategies in these settings.

## Methods

This in-depth analysis of stigma measurement in LIC/LMICs was a secondary analysis of a larger systematic review that explored stigma measurement globally (19). The primary review was registered in PROSPERO (CRD42023433176) and followed PRISMA guidelines. This secondary analysis was not separately registered and was conducted as a *post hoc* analysis of the parent dataset. A medical librarian created search strategies and conducted a comprehensive database search for concepts of scales, instruments, measurements, outcomes, patient stigma, and self-stigma across Embase, Ovid Medline, Scopus, Cochrane Central Register of Controlled Trials, Cochrane Database of Systematic Reviews, Cumulated Index to Nursing and Allied Health, APA PsycInfo, Global Health, and Clinicaltrials.gov. With no limits applied, 14,663 records were returned on the initial search. After de-duplication using EndNote and Covidence.org and two search rounds, the primary review extracted data from 2,267 full-text articles (20, 21). The primary review was designed to characterize how stigma is measured across health conditions and geographic settings globally.

This secondary analysis included full-length, peer-reviewed papers from the primary review if they quantitatively measured stigma surrounding a physical health condition in LICs and/or LMICs. This analysis was undertaken to specifically characterize patterns in stigma literature within LIC and LMIC resource settings. A focused analysis was necessary to further contextualize stigma and its distribution in these settings, where differences in resources, research capacity, and sociocultural contexts may shape and change the way stigma is experienced and studied. LICs and LMICs were defined using the 2025 World Bank classifications regardless of the publication year (22). There were no restrictions on study design or publication dates, and publications had to be written in English, Spanish, or Arabic due to language limitations of the research team. Papers were excluded if they were review/conceptual articles, abstracts only, or conference proceedings. Data was managed using Research Electronic Data Capture (REDCap) tools hosted at Washington University in St. Louis. Studies in the primary review focused on either tool development/validation and/or tool application/use. For this analysis, papers that exclusively focused on tool development were excluded, as they primarily assessed tool metrics rather than categorizing stigma, resulting in 137 publications for final inclusion (Figure 1; Appendix 1). Two authors (BR, VE) extracted additional data on study tools and results specific to this analysis. Data for the first 21 articles (15%) were independently extracted by both authors, with disagreements resolved through consensus. During this process, variable definitions were refined for clarity to support consistency in extraction between reviewers. The remaining studies were extracted by one of the two authors, with ambiguous cases discussed with the second reviewer and resolved through consensus to maintain consistency.

**Figure 1:**
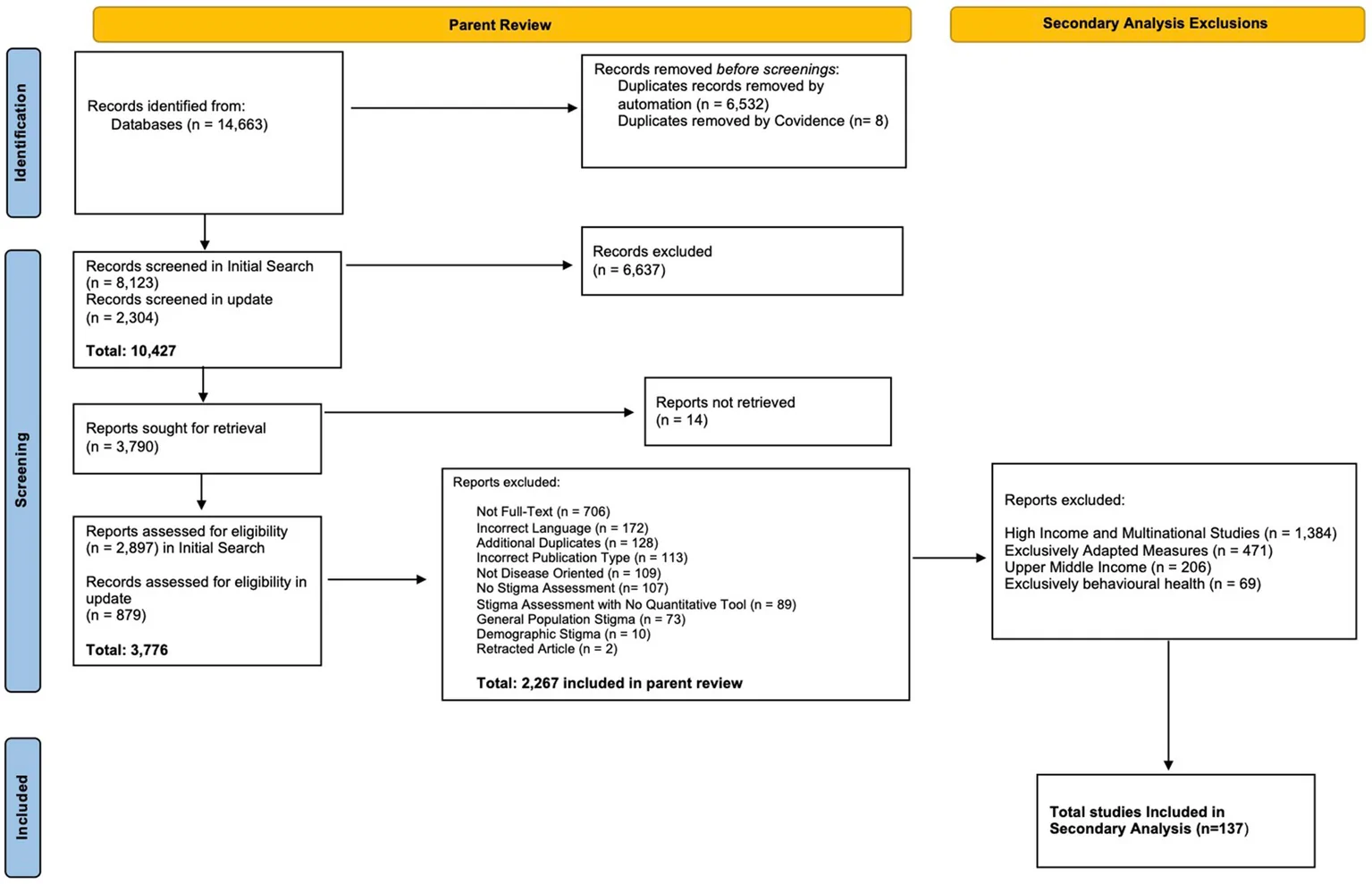
PRISMA diagram.

### Data analysis

Data utilized from the primary review included: World Health Organization (WHO) region and country of each study (23–25), the health condition under investigation, stigma definition, and any interventions designed and implemented. For application studies, sample size, study design, and stigma-focused intervention data were extracted.

For this analysis, additional data extraction included: the reported prevalence of stigma and correlate variables of stigma. We also extracted data related to the construct of stigma studied, such as domains and levels. We defined domains, in accordance with prior literature, as the distinct ways stigma is experienced, categorized as self/internalized stigma, perceived stigma, enacted stigma, or anticipated stigma (26). To describe where stigma operates in society, data was collected regarding the level at which stigma was measured and described by the subjects, and categorized as individual, interpersonal, community, or structural aligning with prior literature (27). Finally, data was collected regarding how stigma was conceptualized for the study (driver, outcome, and mediator). If stigma was described as a contributing factor of the outcome being reported, it was categorized as a driver. If it was considered an impact of the primary measure of the study, stigma was categorized as a reported outcome. If stigma was neither the driver nor the outcome, it was categorized as a mediator. Additionally, for each study, correlations, or characteristics that were significantly associated with an individual’s experience of stigma, were defined. Overall, there were 13 correlation categories. The complete list of variables we included in correlation analysis can be found in Table 1. Finally, physical health conditions were categorized as either communicable or non-communicable in accordance with WHO standards (28). We also collected the efficacy of the intervention data, defined as a statistically significant or otherwise established improvement in the study outcome, that was described the methods of the study. Because outcome measures, intervention types, and follow-up were heterogenous and often descriptive, we did not estimate an overall intervention effect, but rather summarized findings descriptively.

**Table 1:** Correlations.

Correlation			Total *N* = 137
	LIC *N* = 32 (23.4%)	LMIC *N* = 105 (76.6%)	
Age	3	27	30 (21.9%)
Disease severity	3	24	27 (19.7%)
Education	6	20	26 (19.0%)
Employment	1	10	11 (8.0%)
Gender	1	25	26 (19.0%)
Income	0	9	9 (6.6%)
Intrapersonal indicators	9	13	22 (16.1%)
Mental health	21	58	79 (57.7%)
Relationship status	6	21	27 (19.7%)
Religion	1	4	5 (3.6%)
Social/cultural structure	9	16	25 (18.2%)
Treatment outcome	7	14	21 (15.3%)
Treatment adherence	9	24	33 (24.1%)

To evaluate methodological rigor, a quality appraisal of each paper was conducted using nine questions with dichotomous yes or no scoring (Table 2). Due to the heterogeneity of studies and study designs, no single quality appraisal could uniformly be applied to satisfy the needs of this study. Instead, tools from Joanna Briggs Institute (29) were used to create a single set of questions that could be applied regardless of study type. Specifically, we included six general methodological items that assessed clarity of inclusion criteria, description of settings and participants, validity and reliability of the stigma measurement, use of statistical analyses, reporting of ethical considerations, and clarity in stigma-related results reporting. Because stigma research poses unique measurement challenges, particularly across diverse cultural contexts, three questions (6,7,8) were added to evaluate the rigor of stigma-specific aspects of these studies, including clarity in stigma conceptualization, the validation of the stigma measurement within the population, and if the study explicitly considered the cultural relevance of stigma or its measurement. These criteria were added because stigma is a context-dependent construct, where meaning and manifestations of stigma can vary across health conditions, populations and sociocultural settings. These factors can substantially affect interpretation and comparability of stigma estimates across settings (12, 30). This appraisal was intended to characterize the methodological strengths and limitation across our studies rather than generate a validated summary score for each study.

**Table 2:** Quality appraisal.

Questions	Total papers *N* = 137	LICs *N* = 32	LMICs *N* = 105
1. Was the criteria for inclusion in the sample clearly defined?	131 (95.6%)	32 (100.0%)	99 (94.3%)
2. Were the study participants and setting described in detail?	137 (100%)	32 (100.0%)	105 (100%)
3. Was stigma measured in a valid and reliable way?	83 (60.6%)	18 (56.3%)	65 (61.9%)
4. Were statistical analyses used (more than descriptive statistics)?	134 (97.8%)	32 (100.0%)	102 (97.1%)
5. Were ethical considerations described? (Intro/Con)	123 (90.5%)	30 (93.8%)	93 (88.6%)
6. Was stigma clearly conceptualized and defined?	101 (73.7%)	16 (50.0%)	85 (81.0%)
7. Was the stigma measure validated for this population?	40 (29.2%)	12 (37.5%)	28 (26.7%)
8. Did the study consider cultural relevance of stigma?	58 (42.3%)	15 (46.9%)	43 (41.0%)
9. Were results of stigma clearly presented in results?	135 (98.5%)	32 (100.0%)	103 (98.1%)

Analyses were descriptive in nature and univariate statistics were used to summarize study characteristics, stigma definitions, stigma constructs, and outcome measures. Meta-analysis was not feasible due to the heterogeneity across studies.

## Results

Overall, 137 papers were included in this analysis (Figure 1). Most studies (*n* = 105, 76.6%) were conducted in LMICs with the remaining (*n* = 32, 23.4%) in LICs. Studies exclusively focused on the adult population predominated (*n* = 92/137, 67.2%), with 22 of the LIC papers (22/32, 68.8%) and 70 of the LMIC papers (70/105, 66.7%) focused on adult populations. Only eight papers (5.8%) measured stigma exclusively in pediatric populations, and 24 papers (17.5%) studied both adult and pediatric populations. Studies were conducted across all WHO regions, except the European region, which contains no LICs and only three LMICs (Figure 2). Globally, most LICs and many LMICs are in the African WHO region. Correspondingly, the African region was the most represented in our review, with all but one LIC paper (*n* = 31/32, 96.9%) and almost half of the papers from LMICs (*n* = 42/105, 40.0%) coming from the African region. The remaining LIC paper came from Yemen in the Eastern Mediterranean region. LMICs in the Southeast Asia region were also well represented within the review (*n* = 35/105, 33.3%). Additional regional data is provided in Table 3.

**Figure 2:**
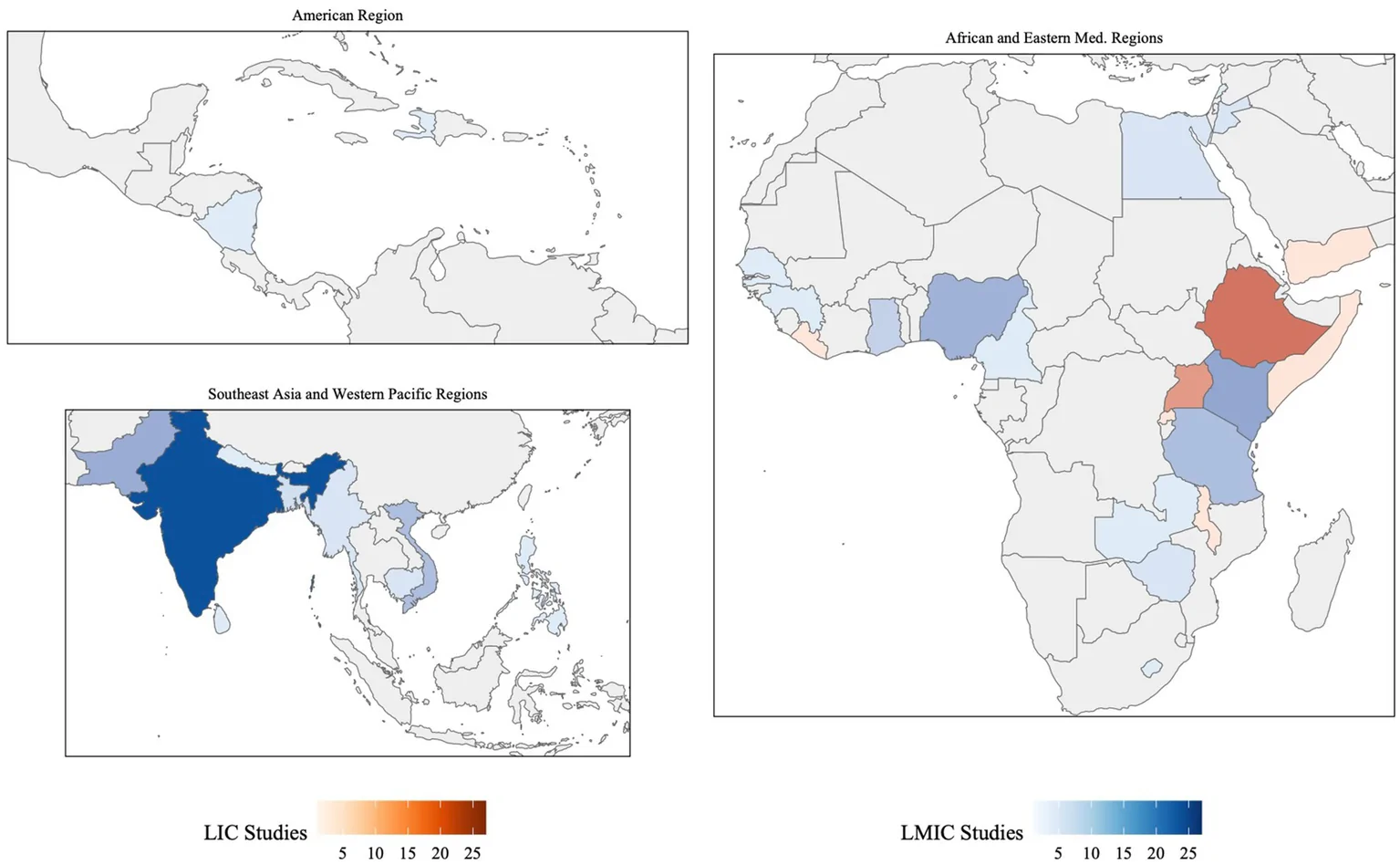
Number of studies by country and income group.

**Table 3:** Study characteristics stratified by LIC or LMIC (*N* = 137 papers).

	LIC papers *N* = 32 (23.4%)	LMIC papers *N* = 105 (76.6%)
Population of interest		
Adult (18+)	22 (68.8%)	70 (66.7%)
Pediatric (<18)	2 (6.3%)	6 (5.7%)
Both	7(21.9%)	17 (16.2%)
Not specified	1 (3.1%)	12 (11.4%)
Respondent		
Patient Stigma	31 (96.9%)	100 (95.2%)
Patient Proxy	0 (0.0%)	1 (1.0%)
Caregiver Stigma	1 (3.1%)	8 (7.6%)
Total participants		
0–100	4 (12.5%)	16 (15.2%)
101–250	7 (21.9%)	46 (43.8%)
251–500	19 (59.4%)	21 (20.0%)
501–1,000	2 (6.3%)	12 (11.4%)
1,001 – 2,000	0 (0.0%)	9 (8.6%)
2,001+	0 (0.0%)	1 (1.0%)
Number of stigma tools used		
1	30 (93.8%)	91 (86.7%)
2	0 (0.0%)	10 (9.5%)
3+	2 (6.3%)	4 (3.8%)
WHO Region		
African	31 (96.9%)	42 (40.0%)
Region of Americas	0 (0.0%)	2 (1.9%)
South-East Asia	0 (0.0%)	35 (33.3%)
European	0 (0.0%)	0 (0.0%)
Eastern Mediterranean	1 (3.1%)	16 (15.2%)
Western pacific	0 (0.0%)	10 (9.5%)
Health condition*		
*Some studies analyzed multiple health conditions and double counted		
**Conditions such as arthritis, metabolic syndrome and podoconiosis		
HIV	20 (62.5%)	61 (58.1%)
Epilepsy	5 (15.6%)	9 (8.6%)
Parkinsons	0 (0.0%)	2 (1.9%)
Leprosy	1 (3.1%)	7 (6.7%)
Obesity	0 (0.0%)	3 (2.9%)
Dermatological conditions	1 (3.1%)	5 (4.8%)
TB	2 (6.3%)	9 (8.6%)
COVID-19	1 (3.1%)	4 (3.8%)
Cancer	0 (0.0%)	4 (3.8%)
T1DM	0 (0.0%)	1 (1.0%)
Asthma/COPD	0 (0.0%)	1 (1.0%)
Other**	3 (9.4%)	2 (1.9%)
Study design		
Cross-sectional	21 (65.6%)	82 (78.1%)
Randomized study	4 (12.5%)	8 (7.6%)
Case–control	3 (9.4%)	3 (2.9%)
Longitudinal	1 (3.1%)	3 (2.9%)
Mixed methods	1 (3.1%)	3 (2.9%)
Pre and post-test	0 (0.0%)	4 (3.8%)
Non-randomized experimental Treatment/intervention	1 (3.1%)	2 (1.9%)
Prospective cohort	1 (3.1%)	0 (0.0%)

Stigma studies from LICs and LMICs predominantly focused on communicable diseases (*n* = 104/137, 75.9%), specifically HIV (*n* = 81/137, 59.1%) (Figure 3). This was true in LICs, where the most frequently studied communicable diseases were HIV (*n* = 20/32, 62.5%), tuberculosis (TB) (*n* = 2/32, 6.3%), leprosy (*n* = 1/32, 3.1%), and COVID-19 (*n* = 1/32, 3.1%), and epilepsy was the most studied non-communicable disease (*n* = 5/32, 15.6%). In LMICs, a similar proportion of studies focused on communicable diseases (*n* = 80/105, 76.2%). Studies from LMICs also included those exploring stigma in a wider variety of non-communicable diseases such as dermatologic conditions (*n* = 5/105, 4.8%), cancer (*n* = 4/105, 3.8%), obesity (*n* = 3/105, 2.9%), asthma (*n* = 1/105, 1.0%), and Type 1 Diabetes Mellitus (T1DM) (*n* = 1/105, 1.0%) (Table 4).

**Figure 3:**
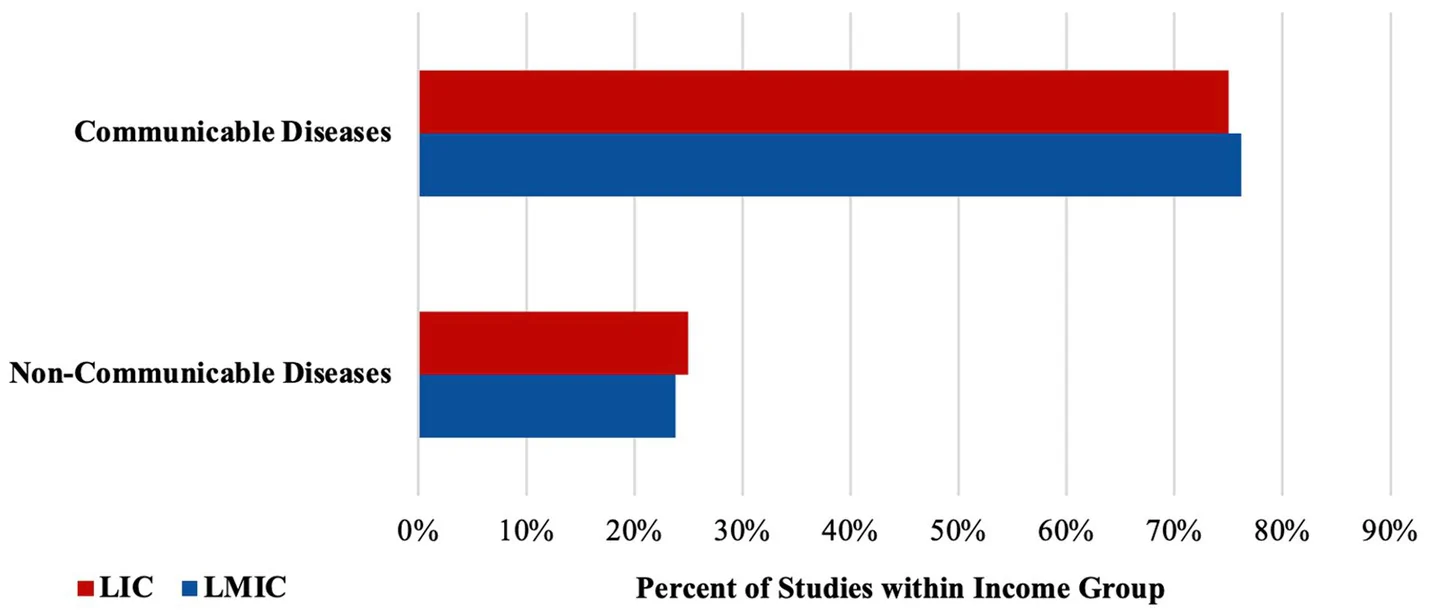
Disease type by income group.

**Table 4:** Health conditions categorized into region*.

	LIC *N* = 32	LMIC *N* = 105
African Region		
HIV	20	37
Epilepsy	5	3
Leprosy	1	1
Dermatological conditions	1	–
Obesity	–	1
TB	2	1
Cancer	–	1
Other	3	–
Region of Americas		
HIV	–	1
TB	–	1
Eastern Mediterranean		
HIV	–	3
Epilepsy	–	3
Obesity	–	1
Dermatological conditions	–	2
TB	–	3
COVID-19	1	2
Other	–	2
Southeast Asia		
HIV	–	12
Epilepsy	–	2
Leprosy	–	6
Obesity	–	1
Dermatological conditions	–	3
TB	–	4
COVID-19	–	2
Cancer	–	3
T1DM	–	1
Asthma/COPD	–	1
Western Pacific		
HIV	–	8
Epilepsy	–	1
Parkinson’s	–	2

*Some studies analyzed multiple health conditions and double counted.

Most studies used a cross-sectional design (*n* = 103/137, 75.2%) and many studies reported on stigma prevalence (*n* = 55/137; 40.1%) (Table 5). However, estimates of stigma prevalence varied widely across studies and were difficult to compare because methods for measuring and reporting stigma were not standardized. Within these unclear reports of stigma (n = 82/137; 59.9%), stigma was described categorically without clear definitions or thresholds for those rankings. Other papers (*n* = 11; 8.0%) reported multiple prevalences within a single paper, for example, providing a prevalence for each domain or level of stigma assessed. Despite the heterogeneity in reporting, of studies reporting stigma prevalence as a percentage, 18 studies (*n* = 18/44, 40.9%) reported >50.0% of their study population experiencing stigma.

**Table 5:** Stigma definitions, prevalence, domain, and levels.

Stigma definition	LIC *N* = 6 (12.8%)	LMIC *N* = 41 (87.2%)	Definitions
Goffman^1^	2 (33.3%)	8 (19.5%)	“The situation of the individual who is disqualified from full social acceptance due to a trait which is deeply discrediting.”
Link and Phelan^2^	1 (16.7%)	3 (7.3%)	“The co-occurrence of labeling, stereotyping, separation, status loss, and discrimination.”
Turan^29^	0 (0.0%)	2 (4.9%)	“The acceptance, acknowledgement and application of negative labels, stereotypes, and societal perceptions.”
Did not use pre-established definition	1 (16.7%)	6 (14.6%)	
Other with one citation	1 (16.7%)	15 (36.6%)	
Other with multiple citations	1 (16.7%)	7 (17.1%)	

	LIC *N* = 32	LMIC *N* = 105
Stigma prevalence		
Report a percent	17 (53.1%)	27 (25.7%)
Report multiple types	1 (3.1%)	10 (9.5%)
Not clearly reported	14 (43.8%)	68 (64.8%)
Stigma domain		
Self/Internalized	13 (40.6%)	62 (59.0%)
Perceived	19 (59.4%)	76 (72.4%)
Enacted	6 (18.8%)	30 (28.6%)
Anticipated	2 (6.3%)	15 (14.3%)
Stigma level		
Individual	24 (75.0%)	90 (85.7%)
Interpersonal	10 (31.3%)	64 (61.0%)
Community	3 (9.4%)	22 (21.0%)
Structural	2 (6.3%)	7 (6.7%)
Stigma usage		
Driver	26 (81.3%)	50 (47.6%)
Outcome	5 (15.6%)	54 (51.4%)
Mediator	1 (3.1%)	1 (1.0%)

### Defining stigma

Most papers (*n* = 90/137, 65.7%) did not explicitly define stigma (Table 5). Of the 47 papers that provided a stigma definition (LIC: *n* = 6/47, 12.8%; LMIC: *n* = 41/47, 87.2%), 40 papers (40/47, 85.1%) used established definitions of stigma, including the Goffman definition (10/40; 25.0%) (1), the Link and Phelan definition (4/40; 10.0%) (2), and the Turan definition (2/40; 5.0%) (31). Some papers provided their own definition of stigma, with no references, which described discriminatory acts, rejection, or exclusion and emphasized stigma being a negative social phenomenon or experience (*n* = 7/47, 14.9%).

### Exploring the conceptualization of stigma

All four stigma domains describing the distinct ways stigma is experienced, (internalized, perceived, enacted, anticipated) were measured, and many papers studied stigma across more than one domain (*n* = 65/137, 47.4%). The most frequently studied domain was perceived stigma (*n* = 95/137, 69.3%), while anticipated stigma was the least measured domain (*n* = 17/137, 12.4%) (Table 5).

Similarly, most papers studied stigma at more than one level (*n* = 79/137, 57.7%). Stigma was most commonly studied at an individual level (*n* = 114/137, 83.2%), followed by interpersonal (*n* = 74/137, 54.0%), community (*n* = 25/137, 18.2%), and structural (*n* = 9, 6.6%) levels. Stigma was studied as the driver, or contributing factor of the outcome being reported, in 55.5% of papers (*n* = 76), while it was studied as an outcome in 43.1% (*n* = 59). In two papers (1.5%) stigma was a mediator, meaning it was neither a driver nor an outcome (Table 5).

### Correlations of stigma

All 137 papers described stigma correlates, within 13 different categories (Table 1). Across all papers, mental health was the most common correlation category (*n* = 79/137, 57.7%) (Table 2). Many studies in both LICs and LMICs demonstrated how stigma around a physical health disorder was associated with poor mental health outcomes. For example, in a study in Ethiopia, higher levels of HIV-related perceived stigma were associated with significantly increased rates of depression (32–36). Similarly, among patients with epilepsy in Pakistan, there was a positive correlation between perceptions of stigma and depression and anxiety (37). In a study of cancer patients in India, elevated internalized stigma was linked with increased anxiety, depression, and hopelessness (36).

The second most common correlate was treatment adherence (*n* = 33/137, 24.1%). Higher stigma measures were found to be associated with poor treatment adherence in many settings. In a study from Ethiopia, patients who experienced higher levels of perceived stigma were more likely to be treatment nonadherent (38). Similarly, in HIV patients in Tanzania, individuals reporting lower internalized HIV stigma demonstrated a greater likelihood of initiating antiretroviral treatment (39).

Patient age was also commonly studied as a correlate (*n* = 30/137, 21.9%), although the ways in which age correlated with stigma were nuanced, with varied patterns across studies. In two studies from Bengal, India, older patients with leprosy and HIV were found to have higher stigma scores (40, 41). In contrast, a study in Kenya found that older HIV patients reported lower levels of internalized stigma (42). Gender was also correlated with stigma (*n* = 25/137, 18.2%), especially in LMICs. In a study of psoriasis patients from India and a study from COVID-19 patients in Egypt, female gender was significantly associated with increased levels of stigma (43, 44).

### HIV study predominance

To assess whether the predominance of HIV studies influenced key aggregate findings of our study, we conducted a stratification analysis comparing HIV (*n* = 81) and non-HIV (*n* = 56) studies (Table 6). Several significant differences emerged. HIV studies more frequently assessed internalized stigma than non-HIV studies (67.9% vs. 44.7%, *p* ≤ 0.05), whereas perceived stigma was more frequently assessed among non-HIV studies (83.9% vs. 59.3%, *p* ≤ 0.05). Mental health was less frequently examined as a correlate of stigma among HIV than non-HIV studies (50.6% vs. 69.6%, *p* ≤ 0.05). Finally, studies of HIV conditions were less likely to examine stigma as an outcome (38.3% v. 60.7%, *p* ≤ 0.05). No significant HIV versus non-HIV differences were identified for treatment adherence, enacted or anticipated stigma, individual, interpersonal, community, or structural levels of stigma, or stigma examined as a driver or mediator.

**Table 6:** HIV vs. Non-HIV study stratification analysis for key findings.

	HIV *N* = 81 (59.1%)	Non-HIV *N* = 56 (40.9%)	*p*-value of Z score (*significant)
Correlation			
Mental health	41 (50.6%)	39 (69.6%)	*p* < 0.05*
Treatment adherence	22 (27.2%)	13 (23.2%)	*p* ≥ 0.05
Stigma domain			
Internalized	55 (67.9%)	21 (44.7%)	*p* < 0.05*
Perceived	48 (59.3%)	47 (83.9%)	*p* < 0.05*
Enacted	25 (30.9%)	11 (19.6%)	*p* ≥ 0.05
Anticipated	13 (16.0%)	5 (8.9%)	*p* ≥ 0.05
Stigma level			
Individual	71 (87.7%)	49 (87.5%)	*p* ≥ 0.05
Interpersonal	44 (54.3%)	34 (60.7%)	*p* ≥ 0.05
Community	18 (22.2%)	7 (13.0%)	*p* ≥ 0.05
Structural	6 (7.4%)	3 (5.4%)	*p* ≥ 0.05
Stigma usage			
Driver	56 (69.1%)	46 (82.1%)	*p* ≥ 0.05
Outcome	31 (38.3%)	34 (60.7%)	*p* < 0.05*
Mediator	1 (1.2%)	1 (1.8%)	*p* ≥ 0.05

Intervention efficacy	HIV *N* = 13 (72.2%)	Non-HIV *N* = 5 (27.8%)	*p*-value of Z score (*significant)
Effective	8 (61.5%)	5 (100.0%)	*p* ≥ 0.05
Non-effective	5 (38.5%)	0 (0.00%)	*p* ≥ 0.05

### Stigma interventions

Only 18 studies (13.1%) evaluated stigma interventions (Table 7). Most of these (*n* = 14/18, 77.8%) were conducted in LMICs. Interventions included support groups with peers and family (45, 46), health education from trained individuals (47), medication distribution (39), cognitive-behavioral therapy (48), and agricultural and financial management aid (49). All interventions targeted the patient. The majority focused on patients with HIV (*n* = 13/18, 72.2%), but others targeted patients with TB (*n* = 2/18, 11.1%), epilepsy (*n* = 1/18, 5.6%), Hepatitis B Virus (*n* = 1/18, 5.6%), and leprosy (*n* = 1/18, 5.6%). Thirteen of these studies (*n* = 13/18, 72.2%) reported effective interventions, demonstrating significant or pre-specified improvement in their study outcome measure. Five studies conducted pre- and post-intervention measures, with no subsequent follow-up (5/18, 27.8%). The longest follow-up period was 2 years (1/18, 5.6%). The rest of the studies had 1 year or less follow-up (12/18, 66.7%). Furthermore, as 13 studies were HIV studies (72.2%), we again conducted a stratification analysis of HIV vs. non-HIV studies, and found no statistical significance in efficacy (*p* > 0.05) (Table 6).

**Table 7:** Interventions.

	LIC *N* = 4 (22.2%)		LMIC *N* = 14 (77.8%)	
	Effective	Not effective	Effective	Not effective
Intervention papers total	3 (75%)	1 (25%)	10 (71.4%)	4 (28.6%)
Intervention types				
Support groups	1	–	4	3
Health education	2	–	2	1
Treatment distribution	–	1	1	–
Other*	–	-	3	–

*Individual counseling, cognitive behavioral therapy, and financial management.

### Quality appraisal

Our quality appraisal showed variability in study quality, but generally acceptable methodological rigor (Table 2). Only 21 of the studies (21/137, 15.3%) scored 100%, while 84 studies (84/137, 61.3%) scored above 70% on the nine-question appraisal. In LICs and LMICs, questions about stigma measure validation and exploring the cultural relevance of stigma had the lowest ratings. Only 40 studies (40/137, 29.2%) reported utilizing a validated stigma measure for study population, and only 58 studies considered the cultural relevance of stigma (58/137, 42.3%).

## Discussion

This systematic review synthesizes existing evidence on stigma in physical health conditions across LICs and LMICs, demonstrating that although stigma is widespread in low resource settings it remains understudied. The existing stigma literature disproportionally reports on communicable diseases, especially HIV, within the African and Southeast Asian WHO regions. Due to multiple and inconsistent stigma definitions and measurement tools, the current evidence remains fragmented. Additionally, the studies lack culturally adapted and population-specific stigma measures. Despite these limitations, our findings reveal multiple constructs that correlate with stigma and could be crucial targets for future stigma reduction strategies.

One of the most striking gaps in the stigma literature from LICs and LMICs is the limited representation of NCDs and pediatric populations. This should be interpreted as a gap in the evidence base that has been identified and not across all settings. The predominance of communicable-disease stigma research in the LIC and LMIC studies likely reflects the high infectious-disease burden in these settings and historical funding priorities for transmissible conditions such as HIV and TB (28, 50, 51). However, NCDs, such as cancer, cardiovascular disease, chronic respiratory disease, and diabetes, account for over 70% of global deaths, with 82% of premature NCD fatalities occurring in Low and Middle Income Countries (28). There are many reasons stigma around non-communicable diseases may be under-studied in LICs and LMICs including less urgency surrounding chronic conditions in these regions, limited research capacity, and fewer dedicated public health response systems (52, 53). Importantly, the small number of NCD studies that have been identified within our evidence base, limits our ability to characterize stigma with the same confidence as for more extensively studied communicable diseases. However, stigma related to NCDs is consequential, especially in LICs and LMICs, where effective management requires sustained, recurrent engagement with often fragmented health care systems (54). This represents a critical gap that could undermine long-term care-seeking, adherence, and health outcomes.

The predominance of HIV (59.1%) within the identified evidence base raises the possibility that overall patterns of physical health-related stigma may disproportionately reflect the established HIV stigma literature. Our stratification analysis showed that HIV studies were significantly more likely to assess internalized stigma, whereas non-HIV studies more frequently assessed perceived stigma, mental health correlates, and stigma as an outcome. These differences suggest that the conceptualization and study of stigma within this evidence base varies across health conditions. Aggregate estimates should not be assumed to characterize physical health-related stigma uniformly across diseases. Consistent with prior literature, shared stigma processes may coexist with condition-specific drivers and manifestations (12). HIV-related stigma, for example, may be shaped by mechanisms related to transmissibility, disclosure, sexual behavior, and social marginalization that may not apply in the same way to cancer, diabetes, epilepsy, or other physical health conditions (55). Importantly, several other dimensions, including enacted and anticipated stigma and the individual, interpersonal, community, and structural levels, did not differ significantly between HIV and non-HIV studies, suggesting some commonality in how stigma has been examined across conditions. Greater representation of noncommunicable diseases is crucial to determine which stigma mechanisms are shared across diseases and which may be more condition specific.

Furthermore, the infrequent representation of pediatric population studies is consistent with findings from the broader stigma literature (19, 56); additionally, the need is even more pronounced in LICs and LMICs, where children face heightened vulnerability due to constrained health systems and limited social protections (15). This lack of pediatric-centered stigma research in these settings potentially obscures the understanding of early life processes that could affect long-term health outcomes (57).

Importantly, aligning with prior reviews, included studies generally adhered to basic methodological standards, but lacked usage of unified, culturally-adapted stigma frameworks and measures (8, 13, 19). Developing standardized, yet flexible approaches to defining and measuring stigma may be particularly valuable in LICs and LMICs, where research capacity constraints increase the need for comparable, efficient, and contextually valid tools (5, 27). Additionally, the implications of heterogeneity may be more consequential where inconsistencies hinder comparability of stigma prevalence and implementation of interventions across settings with diverse languages, cultures, regions, and health systems. The differences in reported stigma prevalence could reflect a variation in how stigma was conceptualized and measured, including scoring thresholds, translation, cultural adaptation, or even instrument content, rather than true differences in stigma burden. This is particularly relevant for cross-country comparisons that may be done in the future where the same instrument may not capture the social specific aspects of stigma consistently across populations.

Structural stigma was captured in only a small proportion of studies identified in this analysis. Structural stigma can be difficult to capture with patient-reported stigma measures, which were the focus of the primary systematic review. Therefore, our finding of limited structural-level studies in this analysis could be a limited representation within the identified literature, rather than evidence that structural stigma itself being uncommon in LIC/LMICs.

Studies consistently demonstrated strong associations between stigma and mental health outcomes as well as poor treatment adherence. These findings are aligned with a prior review demonstrating that stigma harms psychosocial well-being and hinders help-seeking in limited resource contexts (51). In LICs and LMICs, there is often limited access to mental health services and follow-up care, which could magnify the adverse effects of stigma (58–60). Additional associations, including age, were more nuanced. It is possible that this nuance is related to the heath condition studied. For example, studies have shown that some patients with HIV learn to live with their disease and build life experience and social support overtime, which may buffer the stigma they experience as they age (61). There are also likely regional and cultural factors influencing the age-stigma association. For example, South Asian and African regions are more age-reverent regions, which could buffer the stigma experienced in older populations (62, 63). Demographic correlative findings, such as age and gender, should be further examined in future work as they may provide insights regarding vulnerable populations.

Although relatively few studies evaluated stigma-reduction interventions, most that did reported short-term effectiveness, particularly those incorporating psychosocial assistance, counseling, and group-based support (45, 64, 65). These approaches could be promising because they target mechanisms such as internalized stigma, misinformation, social isolation, and resource disparities. This aligns with other studies that aimed to reduce stigma across HIV and mental health conditions, which have similarly used counseling, social support, and health education to address stigma at individual and interpersonal levels (13, 66). However, it is crucial to note that this evidence does not permit comparison of intervention modalities as the predominance of HIV-focused studies limits generalizability to NCDs and other physical conditions. Implementation studies are particularly lacking in LICs and LMICs, compared with HICs (12, 13). In low-resource settings most interventions are smaller scale and short-term, leading to less follow-up and sustained stigma reduction (67). Long-term sustainability should be further explored, as prior literature demonstrates that community engagement and integration within existing systems is crucial in allowing implementation work to be sustained and provide long-term impact (8). Bridging this gap requires not only adapting approaches from HICs to the local contexts of LICs and LMICs, but also developing context-specific interventions from the ground up, with a focus on sustainability and investment in local infrastructure.

Key strengths of this study include its broad scope across physical health conditions and focus on vulnerable and understudied populations in LICs and LMICs, as well as rigorous PRISMA-compliant methodology, structured data extraction with dual review for consensus when necessary, and quality appraisal. However, several limitations must be considered. Language restrictions may have resulted in missed studies published in languages outside of the included languages, which could be relevant to this study given the diversity of lingual backgrounds represented in LIC/LMICs. This also could influence conclusions regarding geographic representation itself, as excluding the primary language of specific geographic regions could lead to them being considered under-represented. Furthermore, we used the country-income classification from the 2025 World Bank, regardless of the study publication year. Although this provides a consistent contemporary definition for income settings across the analysis, it does not account for countries that transitioned between income categories during the period represented by our analysis. Future studies could incorporate historical income classifications and examine whether transitions between income categories may influence patterns in distribution and characteristics of stigma research. Additionally, heterogeneity in study designs and measures and a predominance of cross-sectional studies limited comparability and hindered meta-analysis. The pre-dominance of cross-sectional studies also limits the causal inference regarding the associations between stigma and mental health, treatment adherence, or other study outcomes. Furthermore, the primary systematic review was not specifically conducted to comprehensively identify stigma-reduction interventions, structural or provider level stigma, or community level discrimination. The frequency of these topics within our current dataset should be interpreted as the representation among the studies identified within our primary review rather than a comprehensive estimate of the broader literature. Additionally, the frequent use of non-validated or poorly adapted stigma measures could obscure true effect sizes and impacts of stigma. These limitations highlight broader challenges in stigma research in LICs and LMICs and reinforce the need for more rigorous, longitudinal, and context-sensitive study designs.

This review demonstrates that stigma is consistently associated with adverse health outcomes among individuals with physical health conditions in LICs and LMICs, highlighting it importance as a potential consequential and pervasive barrier to positive health outcomes in these settings. Additionally, it reveals critical gaps that limit the ability of current research to inform effective and scalable interventions, while highlighting particularly vulnerable populations in which stigma remains understudied. Standardized and culturally adapted stigma frameworks and measures, as well as investment in long-term stigma interventions to improve patient outcomes in the most limited resource settings are needed.

## Data Availability

The original contributions presented in the study are included in the article/Supplementary material, further inquiries can be directed to the corresponding author/s.

## References

[1] Goffman E. Stigma: Notes on the Management of Spoiled Identity. New York: Touchstone (1986).

[2] Link BG Phelan JC. Conceptualizing stigma. Annu Rev Sociol. (2001) 27:363–85. doi: 10.1146/annurev.soc.27.1.363

[3] Hickling LM Allani S Cella M Scott W. A systematic review with meta-analyses of the association between stigma and chronic pain outcomes. Pain. (2024) 165:1689–701. doi: 10.1097/j.pain.0000000000003243, 38776178 PMC11247453

[4] Rai SS Syurina EV Peters RMH Putri AI Zweekhorst MBM. Non-communicable diseases-related stigma: a mixed-methods systematic review. Int J Environ Res Public Health. (2020) 17:657. doi: 10.3390/ijerph17186657, 32932667 PMC7559120

[5] Earnshaw VA Smith LR Chaudoir SR Amico KR Copenhaver MM. HIV stigma mechanisms and well-being among PLWH: a test of the HIV stigma framework. AIDS Behav. (2013) 17:1785–95. doi: 10.1007/s10461-013-0437-9, 23456594 PMC3664141

[6] Gallimore JB Diaz KG Gunasinghe C Thornicroft G Salisbury TT Gronholm PC. Impact of mental health stigma on help-seeking in the Caribbean: systematic review. PLoS One. (2023) 18:e0291307. doi: 10.1371/journal.pone.0291307, 37699044 PMC10497129

[7] Graetz DE Velasquez T Chitsike I Halalsheh H Cáceres-Serrano A Fuentes L . Stigma in pediatric Cancer: an exploratory study of osteosarcoma and retinoblastoma in Guatemala, Jordan, and Zimbabwe. JCO Glob Oncol. (2024) 10:e2400017. doi: 10.1200/GO.24.00017, 38905576 PMC11191872

[8] Mehta N Clement S Marcus E Stona AC Bezborodovs N Evans-Lacko S . Evidence for effective interventions to reduce mental health-related stigma and discrimination in the medium and long term: systematic review. Br J Psychiatry. (2015) 207:377–84. doi: 10.1192/bjp.bp.114.151944, 26527664 PMC4629070

[9] Fond G Vidal M Joseph M Etchecopar-Etchart D Solmi M Yon DK . Self-stigma in schizophrenia: a systematic review and meta-analysis of 37 studies from 25 high- and low-to-middle income countries. Mol Psychiatry. (2023) 28:1920–31. doi: 10.1038/s41380-023-02003-4 36890299

[10] Dessie ZG Zewotir T. HIV-related stigma and associated factors: a systematic review and meta-analysis. Front Public Health. (2024) 12:1356430. doi: 10.3389/fpubh.2024.1356430, 39109161 PMC11300231

[11] Larkin D Birtle AJ Bradley L Dey P Martin CR Pilkington M . A systematic review of disease related stigmatization in patients living with prostate cancer. PLoS One. (2022) 17:e0261557. doi: 10.1371/journal.pone.0261557, 35148315 PMC8836305

[12] Stangl AL Earnshaw VA Logie CH van Brakel W Simbayi LC Barré I . The health stigma and discrimination framework: a global, crosscutting framework to inform research, intervention development, and policy on health-related stigmas. BMC Med. (2019) 17:31. doi: 10.1186/s12916-019-1271-3, 30764826 PMC6376797

[13] Kemp CG Jarrett BA Kwon CS Song L Jetté N Sapag JC . Implementation science and stigma reduction interventions in low- and middle-income countries: a systematic review. BMC Med. (2019) 17:6. doi: 10.1186/s12916-018-1237-x, 30764820 PMC6376798

[14] Paterson A Cheyne A Jones B Schilling S Sigfrid L Stolow J . Systematic review of scales for measuring infectious disease–related stigma. Emerg Infect Dis. (2024) 30:519–29. doi: 10.3201/eid3003.230934, 38407230 PMC10902531

[15] Spencer N Raman S O’Hare B Tamburlini G. Addressing inequities in child health and development: towards social justice. BMJ Paediatr Open. (2019) 3:e000503. doi: 10.1136/bmjpo-2019-000503 PMC6688679 31423469

[16] Saeed F Mihan R Mousavi SZ Reniers RLEP Bateni FS Alikhani R . A narrative review of stigma related to infectious disease outbreaks: what can be learned in the face of the Covid-19 pandemic? Front Psych. (2020) 11:565919. doi: 10.3389/fpsyt.2020.565919, 33343414 PMC7738431

[17] Hajat C Stein E. The global burden of multiple chronic conditions: a narrative review. Prev Med Rep. (2018) 12:284–93. doi: 10.1016/j.pmedr.2018.10.008, 30406006 PMC6214883

[18] Aitambayeva N Aringazina A Nazarova L Faizullina K Bapayeva M Narymbayeva N . A systematic review of tuberculosis stigma reduction interventions. Healthcare (Basel). (2025) 13:1846. doi: 10.3390/healthcare13151846, 40805879 PMC12346600

[19] Malone S Counts L Zabotka L Williams A Loecher N Wynja K . Stigma measurement in health: a systematic review. EClinicalMedicine. (2025) 86:103360. doi: 10.1016/j.eclinm.2025.103360, 40747368 PMC12311962

[20] Covidence. *Better systematic Review Management*. Covidence. (2025). Available online at: https://www.covidence.org/ (Accessed July 25, 2025).

[21] EndNote. *The Best Citation and Reference Management Tool*. EndNote. (2026). Available online at: https://endnote.com/ (Accessed April 22, 2026).

[22] World Bank. *World Bank Country and Lending Groups*. World Bank Data Help Desk. (2026). Available online at: https://datahelpdesk.worldbank.org/knowledgebase/articles/906519-world-bank-country-and-lending-groups (Accessed February 22, 2026).

[23] World Health Organization. *World Regions According to the World Health Organization. Our World in Data*. Geneva: World Health Organization (2026). Available online at: https://ourworldindata.org/grapher/who-regions (Accessed February 22, 2026).

[24] Harris PA Taylor R Minor BL Elliott V Fernandez M O’Neal L . The REDCap consortium: building an international community of software platform partners. J Biomed Inform. (2019) 95:103208. doi: 10.1016/j.jbi.2019.103208, 31078660 PMC7254481

[25] Harris PA Taylor R Thielke R Payne J Gonzalez N Conde JG. Research electronic data capture (REDCap)--a metadata-driven methodology and workflow process for providing translational research informatics support. J Biomed Inform. (2009) 42:377–81. doi: 10.1016/j.jbi.2008.08.010, 18929686 PMC2700030

[26] Pescosolido BA Martin JK. The stigma complex. Annu Rev Sociol. (2015) 41:87–116. doi: 10.1146/annurev-soc-071312-145702, 26855471 PMC4737963

[27] Hatzenbuehler ML. Structural stigma and health inequalities: research evidence and implications for psychological science. Am Psychol. (2016) 71:742–51. doi: 10.1037/amp0000068, 27977256 PMC5172391

[28] WHO. *Our Work: Communicable and Noncommunicable Diseases, and Mental Health*. Available online at: https://www.who.int/our-work/communicable-and-noncommunicable-diseases-and-mental-health (Accessed July 24, 2025).

[29] JBI. *JBI Critical Appraisal Tools*. JBI. Available online at: https://jbi.global/critical-appraisal-tools (Accessed July 25, 2025).

[30] Mokkink LB Terwee CB Patrick DL Alonso J Stratford PW Knol DL . The COSMIN checklist for assessing the methodological quality of studies on measurement properties of health status measurement instruments: an international Delphi study. Qual Life Res. (2010) 19:539–49. doi: 10.1007/s11136-010-9606-8, 20169472 PMC2852520

[31] Turan B Budhwani H Fazeli PL Browning WR Raper JL Mugavero MJ . How does stigma affect people living with HIV? The mediating roles of internalized and anticipated HIV stigma in the effects of perceived community stigma on health and psychosocial outcomes. AIDS Behav. (2017) 21:283–91. doi: 10.1007/s10461-016-1451-5, 27272742 PMC5143223

[32] Duko B Toma A Asnake S Abraham Y. Depression, anxiety and their correlates among patients with HIV in South Ethiopia: an institution-based cross-sectional study. Front Psych. (2019) 10:290. doi: 10.3389/fpsyt.2019.00290, 31133893 PMC6514188

[33] Geta ET Guteta TO Tiruneh G. Impairment of health-related quality of life and its determinants among patients with podoconiosis in east Wollega zone, Oromia regional state, Ethiopia: institutional-based cross-sectional study. BMJ Open. (2024) 14:e077268. doi: 10.1136/bmjopen-2023-077268, 38553080 PMC10982751

[34] Kumar N Unnikrishnan B Thapar R Mithra P Kulkarni V Holla R . Stigmatization and discrimination toward people living with HIV/AIDS in a Coastal City of South India. J Int Assoc Provid AIDS Care. (2017) 16:226–32. doi: 10.1177/2325957415569309, 25656862

[35] Thomford NE Dampson FN Adjei G Eliason S Ekor M Kyei GB. Psychometric assessment of HIV stigma in patients attending a tertiary facility: an initial validation of the Berger HIV stigma scale in a Ghanaian perspective. PLoS One. (2023) 18:e0282193. doi: 10.1371/journal.pone.0282193, 37104472 PMC10138471

[36] Squiers L Siddiqui M Kataria I Dhillon PK Aggarwal A Bann C . Perceived, Experienced, and Internalized Cancer Stigma: Perspectives of Cancer Patients and Caregivers in India. Triangle Park, NC: RTI Press (2021). 35107913

[37] Malik NI Fatima R Ullah I Atta M Awan A Nashwan AJ . Perceived stigma, discrimination and psychological problems among patients with epilepsy. Front Psych. (2022) 13:870. doi: 10.3389/fpsyt.2022.1000870, 36440406 PMC9681901

[38] Getnet A Woldeyohannes SM Bekana L Mekonen T Fekadu W Menberu M . Antiepileptic drug nonadherence and its predictors among people with epilepsy. Behav Neurol. (2016) 2016:1–6. doi: 10.1155/2016/3189108, 28053370 PMC5178347

[39] Tun W Apicella L Casalini C Bikaru D Mbita G Jeremiah K . Community-based antiretroviral therapy (ART) delivery for female sex workers in Tanzania: 6-month ART initiation and adherence. AIDS Behav. (2019) 23:142–52. doi: 10.1007/s10461-019-02549-x, 31197700 PMC6773663

[40] Bandyopadhyay A Samanta S Mukherjee A Bhattacherjee S. Assessing the prevalence and socio-demographic determinants of the unsolved problem of perceived social stigma: a study among leprosy-affected persons in North Bengal, India. Med J Babylon. (2022) 19:258–64. doi: 10.4103/MJBL.MJBL_24_22

[41] Adhikari K Dutt D Pal D Hazra S. Stigma and its associated factors among people living with HIV/AIDS (PLHA) attending ART centre in a tertiary care institute in Kolkata. MAMC J Med Sci. (2022) 8:62–9. doi: 10.4103/mamcjms.mamcjms_87_21

[42] Yator O Mathai M Albert T Kumar M. Burden of HIV-related stigma and post-partum depression: a cross-sectional study of patients attending prevention of mother-to-child transmission clinic at Kenyatta National Hospital in Nairobi. Front Psych. (2021) 11:557. doi: 10.3389/fpsyt.2020.532557, 33716799 PMC7947326

[43] Grover S Mehra A Dogra S Hazari N Malhora N Narang T . Internalized stigma and psychiatric morbidity among patients with psoriasis: a study from North India. Indian Dermatol Online J. (2021) 12:97–104. doi: 10.4103/idoj.IDOJ_345_20, 33768029 PMC7982046

[44] Arrab MM Sabola NE ElSalamoni MA Ahmed M Shafik S Doma NI. *Psychological Aspects, Coping Strategies and Perceived Social Stigma Associated with COVID-19 Pandemic*. (2024).

[45] Alinaitwe R Seggane M Turiho A Bird V Priebe S Sewankambo N. Family psycho-social involvement intervention for severe mental illness in Uganda. S Afr J Psychiatry. (2024) 30:2138. doi: 10.4102/sajpsychiatry.v30i0.2138, 38322180 PMC10839168

[46] Macq J Solis A Martinez G Martiny P. Tackling tuberculosis patients’ internalized social stigma through patient centred care: an intervention study in rural Nicaragua. BMC Public Health. (2008) 8:154. doi: 10.1186/1471-2458-8-154, 18466604 PMC2396624

[47] Bogart LM Matovu JKB Wagner GJ Green HD Storholm ED Klein DJ . A pilot test of game changers, a social network intervention to empower people with HIV to be prevention advocates in Uganda. AIDS Behav. (2020) 24:2490–508. doi: 10.1007/s10461-020-02806-4, 32030525 PMC7415589

[48] Abbas Q Nisa M Khan MU Anwar N Aljhani S Ramzan Z . Brief cognitive behavior therapy for stigmatization, depression, quality of life, social support and adherence to treatment among patients with HIV/AIDS: a randomized control trial. BMC Psychiatry. (2023) 23:539. doi: 10.1186/s12888-023-05013-2, 37491185 PMC10367308

[49] Weiser SD Sheira LA Weke E Zakaras JM Wekesa P Frongillo EA . An agricultural livelihood intervention is associated with reduced HIV stigma among people with HIV. AIDS. (2024) 38:1553–9. doi: 10.1097/QAD.0000000000003928, 38752558 PMC12439198

[50] Boutayeb A. "The Burden of Communicable and Non-Communicable Diseases in Developing Countries". In: Preedy VR Watson PR, editors. Handbook of Disease Burdens and Quality of Life Measures. Berlin: Springer Verlag. p. 531–46.

[51] Kane JC Elafros MA Murray SM Mitchell EMH Augustinavicius JL Causevic S . A scoping review of health-related stigma outcomes for high-burden diseases in low- and middle-income countries. BMC Med. (2019) 17:17. doi: 10.1186/s12916-019-1250-8, 30764819 PMC6376728

[52] WHO. *Global Action Plan for the Prevention and Control of Noncommunicable Diseases 2013–2020*. (2025). Available online at: https://www.who.int/publications/i/item/9789241506236 (Accessed July 24, 2025).

[53] Hategeka C Adu P Desloge A Marten R Shao R Tian M . Implementation research on noncommunicable disease prevention and control interventions in low- and middle-income countries: a systematic review. PLoS Med. (2022) 19:e1004055. doi: 10.1371/journal.pmed.1004055, 35877677 PMC9359585

[54] Siqueira M Coube M Millett C Rocha R Hone T. The impacts of health systems financing fragmentation in low- and middle-income countries: a systematic review protocol. Syst Rev. (2021) 10:164. doi: 10.1186/s13643-021-01714-5, 34078460 PMC8170990

[55] Mahajan AP Sayles JN Patel VA Remien RH Sawires SR Ortiz DJ . Stigma in the HIV/AIDS epidemic: a review of the literature and recommendations for the way forward. AIDS. (2008) 22:S67–79. doi: 10.1097/01.aids.0000327438.13291.62, 18641472 PMC2835402

[56] Counts LE Tanner RS Chen Y Devidas M Ferrara G Chitsike I . Measuring stigma in pediatric oncology: a cross-sectional analysis of three global sites. JCO Glob Oncol. (2025) 11:e2400213. doi: 10.1200/GO.24.00213, 39778128 PMC11731491

[57] Sales WB Maranhão EF Ramalho CST Macêdo SGGF Souza GF Maciel ÁCC. Early life circumstances and their impact on health in adulthood and later life: a systematic review. BMC Geriatr. (2024) 24:978. doi: 10.1186/s12877-024-05571-4, 39609801 PMC11603629

[58] Joiner AP Tupetz A Peter TA Raymond J Macha VG Vissoci JRN . Barriers to accessing follow up care in post-hospitalized trauma patients in Moshi, Tanzania: a mixed methods study. PLoS Glob Public Health. (2022) 2:e0000277. doi: 10.1371/journal.pgph.0000277, 36962378 PMC10021180

[59] Rathod S Pinninti N Irfan M Gorczynski P Rathod P Gega L . Mental health service provision in low- and middle-income countries. Health Serv Insights. (2017) 10:4350. doi: 10.1177/1178632917694350, 28469456 PMC5398308

[60] WHO. *Mental Health Systems in Selected low- and middle-Income Countries*. Geneva: WHO. (2026). Available online at: https://www.who.int/publications/i/item/9789241547741 (Accessed April 22, 2026).

[61] Psaros C Barinas J Robbins GK Bedoya CA Park ER Safren SA. Reflections on living with HIV over time: exploring the perspective of HIV-infected women over 50. Aging Ment Health. (2015) 19:121–8. doi: 10.1080/13607863.2014.917608, 24903460 PMC4245325

[62] Jahangir S Patil DS Gangopadhyay J Vogt TC. Understanding intergenerational dynamics and social support’s impact on health and well-being of older adults in South Asia: a scoping review. Syst Rev. (2025) 14:86. doi: 10.1186/s13643-025-02833-z, 40217304 PMC11987332

[63] Imafidon E Iyare AE Abudu KU. "Ageing, Ageism, Cultural Representations of the Elderly and the Duty to Care in African Traditions". In: Chimakonam JO Etieyibo E Odimegwu I, editors. Essays on Contemporary Issues in African Philosophy. Cham: Springer (2022)

[64] France NF Macdonald SHF Conroy RR Ferris France N Chiroro P Ni Cheallaigh D . ‘We are the change’ - an innovative community-based response to address self-stigma: a pilot study focusing on people living with HIV in Zimbabwe. PLoS One. (2019) 14:e0210152. doi: 10.1371/journal.pone.0210152, 30759114 PMC6373928

[65] Pollack TM Duong HT Nhat Vinh DT Phuong DT Thuy DH Nhung VTT . A pretest-posttest design to assess the effectiveness of an intervention to reduce HIV-related stigma and discrimination in healthcare settings in Vietnam. J Int AIDS Soc. (2022) 25:e25932. doi: 10.1002/jia2.25932, 35818864 PMC9274370

[66] Majeed T Hopkin G Wang K Nepal S Votruba N Gronholm P . Anti-stigma interventions in low-income and middle-income countries: a systematic review. EClinicalMedicine. (2024) 72:102612. doi: 10.1016/j.eclinm.2024.102612, 38707913 PMC11066569

[67] Brown L Macintyre K Trujillo L. Interventions to reduce HIV/AIDS stigma: what have we learned? AIDS Educ Prev. (2003) 15:49–69. doi: 10.1521/aeap.15.1.49.23844, 12627743

