# Supplementary material for: Exploring stigma of physical health conditions across low and lower-middle income countries: a secondary analysis of a global systematic review
Source: Front Public Health. 2026 Oct 14;14:1912141. doi: 10.3389/fpubh.2026.1912141 (PMC13713842; doi:10.3389/fpubh.2026.1912141)
Supplement: Supplementary file 1 [file Table_1.DOCX]

Appendix 1. Citations of Included Studies

1. Abaynew Y, Deribew A, Deribe K. Factors associated with late presentation to HIV/AIDS care in South Wollo ZoneEthiopia: a case-control study. AIDS Res Ther. 2011;8(1):8. doi:10.1186/1742-6405-8-8

2. Addis B, Wolde M, Minyihun A, Aschalew AY. Prevalence of depression and associated factors among patients with epilepsy at the University of Gondar Comprehensive Specialized Hospital, Northwest Ethiopia, 2019. PLOS ONE. 2021;16(10):e0257942. doi:10.1371/journal.pone.0257942

3. Ashaba C, Musoke D, Wafula ST, Konde-Lule J. Stigma among tuberculosis patients and associated factors in urban slum populations in Uganda. African Health Sciences. 2021;21(4):1640-1650. doi:10.4314/ahs.v21i4.18

4. Cavazos-Rehg P, Byansi W, Doroshenko C, et al. Evaluating potential mediators for the impact of a family-based economic intervention (Suubi+Adherence) on the mental health of adolescents living with HIV in Uganda. Social Science & Medicine. 2021;280:113946. doi:10.1016/j.socscimed.2021.113946

5. Duko B, Toma A, Asnake S, Abraham Y. Depression, Anxiety and Their Correlates Among Patients With HIV in South Ethiopia: An Institution-Based Cross-Sectional Study. Front Psychiatry. 2019;10. doi:10.3389/fpsyt.2019.00290

6. Getnet A, Woldeyohannes SM, Bekana L, et al. Antiepileptic Drug Nonadherence and Its Predictors among People with Epilepsy. Behavioural Neurology. 2016;2016(1):3189108. doi:10.1155/2016/3189108

7. Jaber AAS, Khan AH, Sulaiman SAS, Ahmad N, Anaam MS. Evaluation of Health-Related Quality of Life among Tuberculosis Patients in Two Cities in Yemen. PLOS ONE. 2016;11(6):e0156258. doi:10.1371/journal.pone.0156258

8. Kesande C, Bapolisi A, Kaggwa MM, Nakimuli-Mpungu E, Maling S, Ashaba S. Prevalence and factors associated with psychological distress among pregnant and non-pregnant youth living with HIV in rural Uganda: a comparative study. Psychology, Health & Medicine. 2023;28(2):344-358. doi:10.1080/13548506.2022.2050270

9. Kirabira J, Ashaba S, Favina A, Maling S, Nansera D, Zanoni BC. Intrapersonal predictors of internalized stigma among school going adolescents living with HIV in southwestern Uganda. medRxiv. Preprint posted online June 2, 2022:2022.06.01.22275873. doi:10.1101/2022.06.01.22275873

10. Lifson AR, Workneh S, Hailemichael A, Demissie W, Slater L, Shenie T. Perceived social support among HIV patients newly enrolled in care in rural Ethiopia. AIDS Care. 2015;27(11):1382-1386. doi:10.1080/09540121.2015.1098765

11. Moges NA, Adesina OA, Okunlola MA, Berhane Y, Akinyemi JO. Psychological Distress and Its Correlates among Newly Diagnosed People Living with HIV in Northwest Ethiopia: Ordinal Logistic Regression Analyses. Infect Dis (Auckl). 2021;14:1178633721994598. doi:10.1177/1178633721994598

12. Mousley E, Deribe K, Tamiru A, Davey G. The impact of podoconiosis on quality of life in Northern Ethiopia. Health Qual Life Outcomes. 2013;11(1):122. doi:10.1186/1477-7525-11-122

13. Nakandi RM, Kiconco P, Musiimenta A, et al. Understanding patterns of family support and its role on viral load suppression among youth living with HIV aged 15 to 24 years in southwestern Uganda. Health Science Reports. 2022;5(1):e467. doi:10.1002/hsr2.467

14. Strother PJ, Tipayamongkholgul M, Kosaisevee V, Suwannapong N. Effects of psychosocial factors on nonadherence to ART in Ganta, Nimba county, Liberia. AIDS Res Ther. 2022;19(1):27. doi:10.1186/s12981-022-00455-2

15. Vancampfort D, Byansi P, Ward PB, Mugisha J. Correlates of missed HIV appointments in low-resource settings: a study from Uganda. African Journal of AIDS Research. 2021;20(2):125-131. doi:10.2989/16085906.2021.1895235

16. Vancampfort D, Byansi P, Kinyanda E, Bbosa RS, Mugisha J. Internalised HIV-related stigma associated with physical inactivity in people with HIV and AIDS: A cross-sectional study from Uganda. African Journal of AIDS Research. 2021;20(3):238-243. doi:10.2989/16085906.2021.1979607

17. Wagner GJ, Gwokyalya V, Akena D, et al. Stressors and Maladaptive Coping Mechanisms Associated with Elevated Perinatal Depressive Symptoms and Suicidality Among Women Living with HIV in Uganda. IntJ Behav Med. 2023;30(5):743-752. doi:10.1007/s12529-022-10124-3

18. Yimer S, Girma Z, Mengistu N, Shumye S. Determinants of Common Mental Disorders Among People Living with HIV/AIDS Attending ART Service in Southern Ethiopia. HIV. 2021;13:917-925. doi:10.2147/HIV.S329256

19. Yimer Tawiye N, Mekonnen Assefa Z, Gizeyatu Zengye A. Patient satisfaction and associated factors among adults attending ART clinic at Dessie refferal Hospital, Amhara Region, Ethiopia. International Journal of Africa Nursing Sciences. 2021;14:100297. doi:10.1016/j.ijans.2021.100297

20. Yousuf A, Musa R, Isa MLM, Arifin SRM. Anxiety and Depression Among Women Living with HIV: Prevalence and Correlations. doi:10.2174/1745017902016010059

21. Zewude SB, Ajebe TM. Magnitude of optimal adherence and predictors for a low level of adherence among HIV/AIDS-infected adults in South Gondar zone, Northwest Ethiopia: a multifacility cross-sectional study. BMJ Open. 2022;12(1):e056009. doi:10.1136/bmjopen-2021-056009

22. Alinaitwe R, Seggane M, Turiho A, Bird V, Priebe S, Sewankambo N. Family Psycho-Social Involvement Intervention for severe mental illness in Uganda. South African Journal of Psychiatry. 2024;30(0):10. doi:10.4102/sajpsychiatry.v30i0.2138

23. Bebell LM, Kembabazi A, Musinguzi N, et al. Internalized stigma, depressive symptoms, and the modifying role of antiretroviral therapy: A cohort study in rural Uganda*. SSM - Mental Health. 2021;1:100034. doi:10.1016/j.ssmmh.2021.100034

24. Faraade MH. Effectiveness of Self-stigma Reduction Intervention Program in Improving Psychological Status and Anti-tuberculosis Treatment Adherence Among Tuberculosis Patients of Hargeisa Tuberculosis Hospital - Somalia: A Randomized Controlled Trial. doi:10.1257/rct.12166-1.0

25. Geta ET, Guteta TO, Tiruneh G. Impairment of health-related quality of life and its determinants among patients with podoconiosis in East Wollega Zone, Oromia Regional State, Ethiopia: institutional-based cross-sectional study. BMJ Open. 2024;14(3):e077268. doi:10.1136/bmjopen-2023-077268

26. Gutin SA, Ruark A, Darbes LA, Neilands TB, Mkandawire J, Conroy AA. Supportive couple relationships buffer against the harms of HIV stigma on HIV treatment adherence. BMC Public Health. 2023;23(1):1878. doi:10.1186/s12889-023-16762-w

27. Nurye Y, Tareke M, Tadesse M, Shegaw M, Mekonen T. Depression among people with chronic skin disease at Boru Meda Hospital in Northeast Ethiopia. PLOS ONE. 2023;18(2):e0282022. doi:10.1371/journal.pone.0282022

28. Solomon Y, Teshome Y, Ejigu S, Bezabih M. Prevalence of anti-seizure medication nonadherence and its associated factors, among people with epilepsy in North Shewa, Ethiopia, 2021. Epilepsy Behav. 2023;145. doi:10.1016/j.yebeh.2023.109301

29. Yenealem B, Negash M, Madoro D, et al. Prevalence and associated factors of maternal depression among mothers of children with undernutrition at comprehensive specialized hospitals in Northwest Ethiopia in 2023: a cross−sectional study. Front Psychiatry. 2024;15. doi:10.3389/fpsyt.2024.1400293

30. Yesuf W, Hiko D, Alemayehu E, Kusheta S, Shita A, Beyene M. Health-related quality of life in epilepsy and its associated factors among adult patients with epilepsy attending Mizan Tepi University Teaching Hospital, Southwest Ethiopia: a cross-sectional study. BMJ Open. 2024;14(1):e079165. doi:10.1136/bmjopen-2023-079165

31. Bogart LM, Matovu JKB, Wagner GJ, et al. A Pilot Test of Game Changers, a Social Network Intervention to Empower People with HIV to be Prevention Advocates in Uganda. AIDS Behav. 2020;24(9):2490-2508. doi:10.1007/s10461-020-02806-4

32. Hill SE, Zhang C, Remera E, et al. Association Between Clinical Encounter Frequency and HIV-Related Stigma Among Newly-Diagnosed People Living with HIV in Rwanda. AIDS Behav. 2024;28(4):1390-1400. doi:10.1007/s10461-023-04226-6

33. Adjei P, Nkromah K, Akpalu A, et al. A cross-sectional comparative study of perceived stigma between patients with epilepsy and patients living with HIV/AIDS in Accra, Ghana. Epilepsy Behav. 2018;89:1-7. doi:10.1016/j.yebeh.2018.10.015

34. Akhtar N, Batool I. Social stigma perceived by patients of tuberculosis: Gender, age and marital status based differences. 2021;46(3).

35. Aung S, Hardy N, Chrysanthopoulou S, et al. Evaluation of peer-to-peer HIV counseling in Myanmar: a measure of knowledge, adherence, and barriers. AIDS Care. 2022;34(6):762-770. doi:10.1080/09540121.2021.1902929

36. Bandyopadhyay A, Samanta S, Mukherjee A, Bhattacherjee S. Assessing the Prevalence and Socio-Demographic Determinants of the Unsolved Problem of Perceived Social Stigma: A Study among Leprosy-Affected Persons in North Bengal, India. Medical Journal of Babylon. 2022;19(2):258. doi:10.4103/MJBL.MJBL_24_22

37. Barker D, Enimil A, Galárraga O, et al. In-Clinic Adolescent Peer Group Support for Engagement in Sub-Saharan Africa: A Feasibility and Acceptability Trial. J Int Assoc Provid AIDS Care. 2019;18:2325958219835786. doi:10.1177/2325958219835786

38. Bhwana D, Das L, Siewe Fodjo JN, et al. A peer support group intervention to decrease epilepsy-related stigma in an onchocerciasis-endemic area in Mahenge, Tanzania: A pilot study. Epilepsy & Behavior. 2021;124:108372. doi:10.1016/j.yebeh.2021.108372

39. Dalky HF, Gharaibeh H, Faleh R. Psychosocial Burden and Stigma Perception of Jordanian Patients With Epilepsy. Clin Nurs Res. 2019;28(4):422-435. doi:10.1177/1054773817747172

40. Dow DE, Turner EL, Shayo AM, Mmbaga B, Cunningham CK, O’Donnell K. Evaluating mental health difficulties and associated outcomes among HIV-positive adolescents in Tanzania. AIDS Care. 2016;28(7):825-833. doi:10.1080/09540121.2016.1139043

41. Eguzo K, Oluoha C, Onwueyi N, Okon K, Laskar FY. Psychosocial Outcomes and Stigma Among Cancer Patients Undergoing Navigation in Southern Nigeria.

42. Elsayed H, O’Connor C, Leyritana K, Salvana E, Cox SE. Depression, Nutrition, and Adherence to Antiretroviral Therapy in Men Who Have Sex With Men in Manila, Philippines. Front Public Health. 2021;9. doi:10.3389/fpubh.2021.644438

43. France NF, Macdonald SHF, Conroy RR, et al. ‘We are the change’ - An innovative community-based response to address self-stigma: A pilot study focusing on people living with HIV in Zimbabwe. PLOS ONE. 2019;14(2):e0210152. doi:10.1371/journal.pone.0210152

44. Garapati P, Pal B, Siddiqui NA, et al. Knowledge, stigma, health seeking behaviour and its determinants among patients with post kalaazar dermal leishmaniasis, Bihar, India. PLOS ONE. 2018;13(9):e0203407. doi:10.1371/journal.pone.0203407

45. Gaur V, Salvi D, Gautam M, et al. Psychiatric comorbidity in clinically stable COVID-19 patients. Indian Journal of Psychiatry. 2022;64(1):89. doi:10.4103/indianjpsychiatry.indianjpsychiatry_312_21

46. Hamra M, Ross MW, Karuri K, Orrs M, D’Agostino A. The relationship between expressed HIV/AIDS-related stigma and beliefs and knowledge about care and support of people living with AIDS in families caring for HIV-infected children in Kenya. AIDS Care. 2005;17(7):911-922. doi:10.1080/09540120500100593

47. Hamra M, Ross MW, Orrs M, D’Agostino A. Relationship between expressed HIV/AIDS-related stigma and HIV-beliefs/knowledge and behaviour in families of HIV infected children in Kenya. Tropical Medicine & International Health. 2006;11(4):513-527. doi:10.1111/j.1365-3156.2006.01583.x

48. Hosaka KRJ, Mmbaga BT, Shayo AM, et al. A group-based mental health intervention for Tanzanian youth living with HIV: Secondary analysis of a pilot trial. Medicine. 2022;101(7):e28693. doi:10.1097/MD.0000000000028693

49. Jiwanmall SA, Kattula D, Nandyal MB, et al. Weight Stigma in Patients With Obesity and Its Clinical Correlates: A Perspective From an Indian Bariatric Clinic. Cureus. 2022;14. doi:10.7759/cureus.26837

50. Khan FU, Khan FU, Hayat K, et al. Impact of Protracted Displacement on Delay in the Diagnosis Associated with Treatment Outcomes: A Cross-Sectional Study in Internally Displaced Tuberculosis Patients of Pakistan. International Journal of Environmental Research and Public Health. 2021;18(22). doi:10.3390/ijerph182211984

51. Kibria MG, Islam T, Islam MT, Kabir R, Ahmed S, Sultana P. Stigma and its associated factors among patients with COVID-19 in Dhaka City: evidence from a cross-sectional investigation. PeerJ. 2022;10:e14092. doi:10.7717/peerj.14092

52. Kinuthia J, Kohler P, Okanda J, Otieno G, Odhiambo F, John-Stewart G. A community-based assessment of correlates of facility delivery among HIV-infected women in western Kenya. BMC Pregnancy Childbirth. 2015;15(1):46. doi:10.1186/s12884-015-0467-6

53. Kumar N, Unnikrishnan B, Thapar R, et al. Stigmatization and Discrimination toward People Living with HIV/AIDS in a Coastal City of South India. J Int Assoc Provid AIDS Care. 2017;16(3):226-232. doi:10.1177/2325957415569309

54. Kumari P, Ram D, Nizamie SH, Goyal N. Stigma and quality of life in individuals with epilepsy: A preliminary report. Epilepsy Behav. 2009;15(3):358-361. doi:10.1016/j.yebeh.2009.05.003

55. Macq J, Solis A, Martinez G, Martiny P. Tackling tuberculosis patients’ internalized social stigma through patient centred care: An intervention study in rural Nicaragua. BMC Public Health. 2008;8(1):154. doi:10.1186/1471-2458-8-154

56. Malik NI, Fatima R, Ullah I, et al. Perceived stigma, discrimination and psychological problems among patients with epilepsy. Front Psychiatry. 2022;13. doi:10.3389/fpsyt.2022.1000870

57. Mehta KG, Baxi R, Patel S, Chavda P, Mazumdar V. Stigma, Discrimination, and Domestic Violence Experienced by Women Living with HIV: A Cross-sectional Study from Western India. Indian Journal of Community Medicine. 2019;44(4):373. doi:10.4103/ijcm.IJCM_136_19

58. Nazar et al. Psychosocial Predictors of Quality of Life in Patients With Vitiligo. Accessed February 15, 2026. https://pjpr.scione.com/cms/abstract.php?id=21

59. Negi BS, Joshi SK, Nakazawa M, Kotaki T, Bastola A, Kameoka M. Impact of a massive earthquake on adherence to antiretroviral therapy, mental health, and treatment failure among people living with HIV in Nepal. PLOS ONE. 2018;13(6):e0198071. doi:10.1371/journal.pone.0198071

60. Nyandiko W, Chory A, Baum A, et al. Multi-media teacher training and HIV-related stigma among primary and secondary school teachers in Western Kenya. AIDS Care. 2023;35(5):643-650. doi:10.1080/09540121.2022.2119473

61. Oduguwa TO. A comparative study of self stigma between HIV/AIDS and schizophrenia patients. J Psychiatry. 2014;17(2). doi:10.4172/Psychiatry.1000109

62. Ofori-Atta A, Reynolds NR, Antwi S, et al. Prevalence and correlates of depression among caregivers of children living with HIV in Ghana: findings from the Sankofa pediatric disclosure study. AIDS Care. 2019;31(3):283-292. doi:10.1080/09540121.2018.1537463

63. Onono M, Owuor K, Turan J, Bukusi EA, Gray GE, Cohen CR. The Role of Maternal, Health System, and Psychosocial Factors in Prevention of Mother-to-Child Transmission Failure in the Era of Programmatic Scale Up in Western Kenya: A Case Control Study. AIDS Patient Care and STDs. 2015;29(4):204-211. doi:10.1089/apc.2014.0181

64. Onu DU. Treatment adherence mediates the relationship between HIV-related stigma and health-related quality of life. AIDS Care. 2021;33(10):1335-1339. doi:10.1080/09540121.2020.1867701

65. Onyebuchi-Iwudibia O, Brown A. HIV and depression in Eastern Nigeria: The role of HIV-related stigma. AIDS Care. 2014;26(5):653-657. doi:10.1080/09540121.2013.844761

66. Osborn L, Ronen K, Larsen AM, et al. Antenatal depressive symptoms in Kenyan women living with HIV: contributions of recent HIV diagnosis, stigma, and partner violence. AIDS Care. 2022;34(1):69-77. doi:10.1080/09540121.2021.1981216

67. Parcesepe AM, Filiatreau LM, Ebasone PV, et al. Psychiatric comorbidity and psychosocial stressors among people initiating HIV care in Cameroon. PLOS ONE. 2022;17(6):e0270042. doi:10.1371/journal.pone.0270042

68. Pollack TM, Duong HT, Nhat Vinh DT, et al. A pretest-posttest design to assess the effectiveness of an intervention to reduce HIV-related stigma and discrimination in healthcare settings in Vietnam. Journal of the International AIDS Society. 2022;25(S1):e25932. doi:10.1002/jia2.25932

69. Pradhan A, Koirala P, Bhandari SS, et al. Internalized and Perceived Stigma and Depression in Pulmonary Tuberculosis: Do They Explain the Relationship Between Drug Sensitivity Status and Adherence? Front Psychiatry. 2022;13. doi:10.3389/fpsyt.2022.869647

70. Ranjan LK, Gupta PR, Srivastava M. Perceived Stigma and Its Association With Stress, Anxiety, and Depression Among Patients With Epilepsy. The Journal of Nervous and Mental Disease. 2022;210(3):219. doi:10.1097/NMD.0000000000001431

71. Sahu AC, Akhade KS. A cross-sectional study on stigma and discrimination confronted by HIV positive patients in the economic capital of India. Journal of Family Medicine and Primary Care. 2022;11(7):3622. doi:10.4103/jfmpc.jfmpc_2099_21

72. Yakhmi S, Sidhu BS, Kaur B, Dalla EK. STUDY OF HIV RELATED STIGMA IN PEOPLE LIVING WITH HIV/AIDS (PLHA): ROLE OF GENDER DIFFERENCES. Published online 2014.

73. Seb-Akahomen OJ, Lawani AO, James BO. Stigma and suicidality among people living with HIV attending a secondary healthcare facility in Nigeria. Perspectives in Psychiatric Care. 2019;55(4):538-545. doi:10.1111/ppc.12321

74. Smith Fawzi MC, Eustache E, Oswald C, et al. Psychosocial support intervention for HIV-affected families in Haiti: Implications for programs and policies for orphans and vulnerable children. Social Science & Medicine. 2012;74(10):1494-1503. doi:10.1016/j.socscimed.2012.01.022

75. Squiers L, Siddiqui M, Kataria I, et al. Perceived, experienced, and internalized cancer stigma: Perspectives of cancer patients and caregivers in India. RTI Press. Published online April 9, 2021. doi:10.3768/rtipress.2021.rr.0044.2104

76. Swendeman D, Fehrenbacher AE, Roy S, et al. Gender disparities in depression severity and coping among people living with HIV/AIDS in Kolkata, India. PLOS ONE. 2018;13(11):e0207055. doi:10.1371/journal.pone.0207055

77. Sylla M, Vogel AC, Bah AK, et al. Prevalence, severity, and associations of depression in people with epilepsy in Guinea: A single-center study. Epilepsy Behav. 2020;113. doi:10.1016/j.yebeh.2020.107475

78. Taneja N. Home Based Care as an Approach to Improve the Efficiency of treatment for MDR Tuberculosis: A Quasi-Experimental Pilot Study. JCDR. Published online 2017. doi:10.7860/JCDR/2017/27594.10401

79. Thai TT, Tran VB, Nguyen NBT, Bui HHT. HIV-related stigma, symptoms of depression and their association with suicidal ideation among people living with HIV in Ho Chi Minh City, Vietnam. Psychology, Health & Medicine. 2023;28(5):1263-1274. doi:10.1080/13548506.2022.2067342

80. Tran BX, Than PQT, Tran TT, Nguyen CT, Latkin CA. Changing Sources of Stigma against Patients with HIV/AIDS in the Rapid Expansion of Antiretroviral Treatment Services in Vietnam. BioMed Research International. 2019;2019(1):4208638. doi:10.1155/2019/4208638

81. Tran TN, Ha UNL, Nguyen TM, et al. The effect of Non-Motor symptoms on Health-Related quality of life in patients with young onset Parkinson’s Disease: A single center Vietnamese Cross-Sectional study. Clinical Parkinsonism & Related Disorders. 2021;5:100118. doi:10.1016/j.prdoa.2021.100118

82. Tsutsumi A, Izutsu T, Islam MDA, et al. Depressive status of leprosy patients in Bangladesh: association with self-perception of stigma. LEPROSY. 2004;75(1):57-66. doi:10.47276/lr.75.1.57

83. Tsutsumi A, Izutsu T, Md Islam A, Maksuda AN, Kato H, Wakai S. The quality of life, mental health, and perceived stigma of leprosy patients in Bangladesh. Social Science & Medicine. 2007;64(12):2443-2453. doi:10.1016/j.socscimed.2007.02.014

84. Willis N, Milanzi A, Mawodzeke M, et al. Effectiveness of community adolescent treatment supporters (CATS) interventions in improving linkage and retention in care, adherence to ART and psychosocial well-being: a randomised trial among adolescents living with HIV in rural Zimbabwe. BMC Public Health. 2019;19(1):117. doi:10.1186/s12889-019-6447-4

85. Yator O, Mathai M, Vander Stoep A, Rao D, Kumar M. Risk factors for postpartum depression in women living with HIV attending prevention of mother-to-child transmission clinic at Kenyatta National Hospital, Nairobi. AIDS Care. 2016;28(7):884-889. doi:10.1080/09540121.2016.1160026

86. Yator O, Mathai M, Albert T, Kumar M. Burden of HIV-Related Stigma and Post-Partum Depression: A Cross-Sectional Study of Patients Attending Prevention of Mother-to-Child Transmission Clinic at Kenyatta National Hospital in Nairobi. Front Psychiatry. 2021;11. doi:10.3389/fpsyt.2020.532557

87. Yi S, Ngin C, Tuot S, Chhoun P, Fleming T, Brody C. Utilization of traditional, complementary and alternative medicine and mental health among patients with chronic diseases in primary health care settings in Cambodia. Int J Ment Health Syst. 2017;11(1):58. doi:10.1186/s13033-017-0167-x

88. Abbas Q, Nisa M, Khan MU, et al. Brief cognitive behavior therapy for stigmatization, depression, quality of life, social support and adherence to treatment among patients with HIV/AIDS: a randomized control trial. BMC Psychiatry. 2023;23(1):539. doi:10.1186/s12888-023-05013-2

89. Adhikari K, Dutt D, Pal D, Hazra S. Stigma and its Associated Factors among People Living with HIV/AIDS (PLHA) Attending ART Centre in a Tertiary Care Institute in Kolkata. MAMC Journal of Medical Sciences. 2022;8(1):62. doi:10.4103/mamcjms.mamcjms_87_21

90. Afriyie-Adjimi H, Nkyi AK. Impact of body image and perceived stigmatization on the psychological wellbeing of obese women in Kumasi metropolis, Ghana. PLOS ONE. 2024;19(4):e0296061. doi:10.1371/journal.pone.0296061

91. Afzal MI, Jamshaid S, Wang L, et al. Stigmatization, panic disorder, and death anxiety among patients of Covid-19: Fourth wave of pandemic in Pakistan. Acta Psychologica. 2023;236:103924. doi:10.1016/j.actpsy.2023.103924

92. Ahmed S, Méndez RY, Naveed S, et al. Assessment of hepatitis-related knowledge, attitudes, and practices on quality of life with the moderating role of internalized stigma among hepatitis B-positive patients in Pakistan. Health Psychology and Behavioral Medicine. 2023;11(1):2192782. doi:10.1080/21642850.2023.2192782

93. Akoko B, Regan S, Idigbe I, et al. HIV-related stigma and psychological distress in a cohort of patients receiving antiretroviral therapy in Nigeria. AIDS Care. 2024;36(2):204-211. doi:10.1080/09540121.2023.2216006

94. Aliche CJ, Ifeagwazi CM, Ozor CC. Psychological flexibility as a moderator of the relationship between HIV-related stigma and resilience among HIV/AIDS patients. African Journal of AIDS Research. 2022;21(4):345-353. doi:10.2989/16085906.2022.2138473

95. Anjuga Elavarasi E, Smitha MC, Manasa K, Kruthika BN. Depression and stigma experience among patients with tuberculosis in urban and rural settings. Indian Journal of Tuberculosis. 2024;71:S171-S177. doi:10.1016/j.ijtb.2023.03.008

96. Arrab MM, Sabola NE, ElSalamoni MAE, Ahmed M, Shafik S, Doma NI. Psychological Aspects, Coping Strategies and Perceived Social Stigma associated with COVID-19 Pandemic. Published online 2024.

97. Bernard C, Font H, Ziadeh S, et al. Management of depression in people living with HIV/AIDS in Senegal: Acceptability, feasibility and benefits of group interpersonal therapy. Cambridge Prisms: Global Mental Health. 2023;10:e36. doi:10.1017/gmh.2023.31

98. Bibi F, Majeed S, Nikhet S, Makhdoom BJ, Mustafa AB. Disease-Related Stigma, Emotional Regulation and Depression in Patients with HIV. Pakistan Journal of Medical & Health Sciences. 2023;17(11):47-47. doi:10.53350/pjmhs02023171147

99. Bint-e-Saif S, Shahzad S. Importance of perceived social support for HIV/AIDS patients in Pakistan: a collectivist society. Journal of Substance Use. 2024;29(6):1191-1194. doi:10.1080/14659891.2023.2278520

100. Chen WT, Huang F, Shiu CS, et al. Can social support mediate stigma and perceived stress in people live with human immunodeficiency virus? AIDS Care. 2024;36(2):255-262. doi:10.1080/09540121.2023.2254545

101. Gamassa E, Steven E, Mtei R, Kaaya S. Prevalence of Depression and Suicidal Ideation and Associated Risk Factors in Adolescents Receiving Care and Treatment for Hiv/Aids at a Tertiary Health Facility in Kilimanjaro Region, Tanzania. Preprint posted online February 28, 2023. doi:10.21203/rs.3.rs-2534893/v1

102. Goyal AK, Bakshi J, Panda NK, et al. Accuracy of a self-reported Measure in Psychological Assessment when the Instrument is self-administered by the Patient or when Administrated by the Clinician. Indian J Otolaryngol Head Neck Surg. 2023;75(2):1266-1270. doi:10.1007/s12070-022-03379-5

103. Goyal AK, Bakshi J, Panda NK, et al. Shame and Stigma Over Long-Term Survival in Postoperative Cases of Head and Neck Cancer. J Maxillofac Oral Surg. 2024;23(5):1057-1062. doi:10.1007/s12663-023-01931-6

104. Hanson OR, Weglarz AJ, Barabara ML, et al. HIV-related Shame among Women Giving Birth in Tanzania: A Mixed Methods Study. AIDS Behav. 2024;28(7):2276-2285. doi:10.1007/s10461-024-04322-1

105. Hoang VTH, Pham HT, Nguyen LTP, Tran NA, Le-Thi VQT. The relationship between HIV-related stigma and quality of life among HIV infected outpatients: A cross-sectional study in Vietnam. Journal of Public Health Research. 2024;13(1):22799036241238667. doi:10.1177/22799036241238667

106. Kilapilo MS, Mosha IH, Bwire GM, Sambayi GL, Sangeda RZ, Killewo J. Factors Associated with Viral Load Suppression and Indicators of Stigma among People Living with HIV in Dar es Salaam Tertiary Hospitals, Tanzania. Microbiology Research. 2023;14(2):704-713. doi:10.3390/microbiolres14020050

107. Kore S, Bhide A, Bhide V, Shinde A. A Cross Sectional Epidemiological Study to Find the Prevalence of Depression and Anxiety in Patients of Leprosy and It’s Correlation to Stigma Related with Leprosy. International Journal of Pharmaceutical and Clinical Research.

108. Mahfoud D, Fekih-Romdhane F, Abou Zeid J, et al. Functionality appreciation is inversely associated with positive psychotic symptoms in overweight/obese patients with schizophrenia. BMC Psychiatry. 2023;23(1):306. doi:10.1186/s12888-023-04795-9

109. Mangla PM, Barot CK. Stigma and Depression in Patients Suffering from HIV/AIDS. International Journal of Pharmaceutical and Clinical Research.

110. Pratibha, Kavita, Mehta H, Narang T, Singh S. Task sharing for the management of leprosy by nurses in a tertiary healthcare setting of Northern India. Trans R Soc Trop Med Hyg. 2024;118(6):376-383. doi:10.1093/trstmh/trad101

111. Mugo C, Kohler P, Kumar M, et al. Effect of HIV stigma on depressive symptoms, treatment adherence, and viral suppression among youth with HIV. AIDS. 2023;37(5):813. doi:10.1097/QAD.0000000000003473

112. Murshidi R, Hammouri M, Al-Ani A, et al. Investigating the prevalence of body dysmorphic disorder among Jordanian adults with dermatologic and cosmetic concerns: a case–control study. Sci Rep. 2024;14(1):5993. doi:10.1038/s41598-024-56315-8

113. Murugan Y, Patel N, Kumar V, et al. Mental Health Impacts of Multidrug-Resistant Tuberculosis in Patients and Household Contacts: A Mixed Methods Study. Cureus. 2024;16(5). doi:10.7759/cureus.60412

114. Nguyen HLT, Bui TM, Dam VAT, et al. Avoidance of healthcare service use and correlates among HIV-positive patients in Vietnam: a cross-sectional study. BMJ Open. 2023;13(12):e074005. doi:10.1136/bmjopen-2023-074005

115. Sabola NE, Hussien S, Kamel A, et al. Implementing of Nursing Intervention on Knowledge, Perceived Stigma and Health related Outcomes among Patients with Hepatitis B Virus. IJPQA. 2023;14(02):369-376. doi:10.25258/ijpqa.14.2.22

116. Sivaganesh S, Pathmeswaran A. Self-stigma of leprosy affected persons in Jaffna District. Jaffna Medical Journal. 2023;35(2). doi:10.4038/jmj.v35i2.188

117. Tuot S, Sim JW, Nagashima-Hayashi M, et al. What are the determinants of antiretroviral therapy adherence among stable people living with HIV? A cross-sectional study in Cambodia. AIDS Res Ther. 2023;20(1):47. doi:10.1186/s12981-023-00544-w

118. Umar M, Basit A, Ali S, Iqbal Z. Understanding the Burden: Prevalence and Predictors of Depression and Anxiety among Multidrug-Resistant Tuberculosis Patients and their Household Contacts.

119. Verinumbe T, Katomski AS, Turpin G, et al. Characterizing the Relationship between HIV Peer Support Groups and Internalized Stigma Among People Living with HIV in Nigeria. AIDS Behav. 2024;28(3):1068-1076. doi:10.1007/s10461-023-04217-7

120. Weiser SD, Sheira LA, Weke E, et al. An agricultural livelihood intervention is associated with reduced HIV stigma among people with HIV. AIDS. 2024;38(10):1553. doi:10.1097/QAD.0000000000003928

121. Capistrant B, Friedemann-Sánchez G, Pendsey S. Diabetes stigma, parent depressive symptoms and Type-1 diabetes glycemic control in India. Social Work in Health Care. 2019;58(10):919-935. doi:10.1080/00981389.2019.1679321

122. Grover S, Mehra A, Dogra S, et al. Internalized Stigma and Psychiatric Morbidity among Patients with Psoriasis: A Study from North India. Indian Dermatology Online Journal. 2021;12(1):97. doi:10.4103/idoj.IDOJ_345_20

123. Medema-Wijnveen JS, Onono M, Bukusi EA, Miller S, Cohen CR, Turan JM. How Perceptions of HIV-Related Stigma Affect Decision-Making Regarding Childbirth in Rural Kenya. PLOS ONE. 2012;7(12):e51492. doi:10.1371/journal.pone.0051492

124. Tun W, Apicella L, Casalini C, et al. Community-Based Antiretroviral Therapy (ART) Delivery for Female Sex Workers in Tanzania: 6-Month ART Initiation and Adherence. AIDS Behav. 2019;23(2):142-152. doi:10.1007/s10461-019-02549-x

125. Vreeman RC, Scanlon ML, Marete I, et al. Characteristics of HIV-infected adolescents enrolled in a disclosure intervention trial in western Kenya. AIDS Care. 2015;27(sup1):6-17. doi:10.1080/09540121.2015.1026307

126. Badru OA, Babalola OE. Significant Others and Not Family or Friend Support Mediate Between Stigma and Discrimination Among People Living With HIV in Lagos State, Nigeria: A Cross-sectional Study. Journal of the Association of Nurses in AIDS Care. 2023;34(1):96. doi:10.1097/JNC.0000000000000366

127. Gupta N, Samudra M, Dhamija S, Chaudhury S, Saldanha D. Perceived Stigma among Caregivers of Psychiatric Disorders as Compared to Chronic Dermatological Disorders. Medical Journal of Dr DY Patil Vidyapeeth. 2023;16(5):741. doi:10.4103/mjdrdypu.mjdrdypu_916_21

128. Sidhu GS, Garg K, Chopra V. Stigma and self-esteem in patients with bronchial asthma. Monaldi Archives for Chest Disease. 2024;94(3). doi:10.4081/monaldi.2023.2711

129. Thomford NE, Dampson FN, Adjei G, Eliason S, Ekor M, Kyei GB. Psychometric assessment of HIV stigma in patients attending a tertiary facility: An initial validation of the Berger HIV stigma scale in a Ghanaian perspective. PLOS ONE. 2023;18(4):e0282193. doi:10.1371/journal.pone.0282193

130. Ekstrand ML, Heylen E, Mazur A, et al. The Role of HIV Stigma in ART Adherence and Quality of Life Among Rural Women Living with HIV in India. AIDS Behav. 2018;22(12):3859-3868. doi:10.1007/s10461-018-2157-7

131. Elafros MA, Gardiner JC, Sikazwe I, et al. Evaluating layered stigma from comorbid HIV and epilepsy among Zambian adults. eNeurologicalSci. 2018;13:56-62. doi:10.1016/j.ensci.2017.12.001

132. Hayes-Larson E, Hirsch-Moverman Y, Saito S, et al. Depressive symptoms and hazardous/harmful alcohol use are prevalent and correlate with stigma among TB-HIV patients in Lesotho. The International Journal of Tuberculosis and Lung Disease. 2017;21(11):S34-S41. doi:10.5588/ijtld.17.0062

133. Parcesepe AM, Nash D, Tymejczyk O, Reidy W, Kulkarni SG, Elul B. Gender, HIV-Related Stigma, and Health-Related Quality of Life Among Adults Enrolling in HIV Care in Tanzania. AIDS Behav. 2020;24(1):142-150. doi:10.1007/s10461-019-02480-1

134. Suhail K, Chaudhry HR. Impact of perceived fears and stigma on psychosocial problems in patients with epilepsy. Pakistan Journal of Social and Clinical Psychology. 2004;2(1):51-65.

135. Ibikunle PO, Nwokeji SC. Assessment of stigma among people living with Hansen’s disease in south-east Nigeria. Lepr Rev. 2017;88(1):43-57.

136. Van Tam V, Larsson M, Pharris A, et al. Peer support and improved quality of life among persons living with HIV on antiretroviral treatment: a randomised controlled trial from north-eastern Vietnam. Health Qual Life Outcomes. 2012;10:53. doi:10.1186/1477-7525-10-53

137. Than PQT, Tran BX, Nguyen CT, et al. Stigma against patients with HIV/AIDS in the rapid expansion of antiretroviral treatment in large drug injection-driven HIV epidemics of Vietnam. Harm Reduct J. 2019;16(1):6. doi:10.1186/s12954-019-0277-7
